# Succinate dehydrogenase‐deficient malignant paraganglioma complicated by succinate dehydrogenase‐deficient renal cell carcinoma

**DOI:** 10.1002/iju5.12520

**Published:** 2022-08-03

**Authors:** Yoshitomo Yamaguchi, Minato Yokoyama, Akira Takemoto, Yuki Nakamura, Shohei Fukuda, Sho Uehara, Hajime Tanaka, Soichiro Yoshida, Yoh Matsuoka, Yasuhisa Fujii

**Affiliations:** ^1^ Department of Urology Tokyo Medical and Dental University Tokyo Japan; ^2^ Bioresource Research Center Tokyo Medical and Dental University Tokyo Japan

**Keywords:** anaerobic metabolism, paraganglioma, pheochromocytoma, renal cell cancer, succinate dehydrogenase

## Abstract

**Introduction:**

*SDH* Gene mutation is known to be a common cause of pheochromocytoma/paraganglioma and renal cell carcinoma. Here, we report a case of succinate dehydrogenase B‐deficient paraganglioma, which has a high risk of metastasis and recurrence, complicated by succinate dehydrogenase‐deficient renal cell carcinoma, which is rare and accounts for approximately 0.1% of all renal cell carcinomas.

**Case presentation:**

A 50‐year‐old man underwent en bloc resection of a retroperitoneal tumor and the right kidney for retroperitoneal paraganglioma and right renal tumor. Both tumors showed negative expressions of succinate dehydrogenase B in immunostaining. The patient was diagnosed with succinate dehydrogenase‐deficient paraganglioma and succinate dehydrogenase‐deficient renal cell carcinoma. Seventeen months later, retroperitoneal lymphadenectomy revealed lymph node metastasis of the paraganglioma. Deletion of the *SDHB* gene was revealed by genome sequencing of the lymph node.

**Conclusion:**

This is the first reported case of synchronously diagnosed succinate dehydrogenase‐deficient paraganglioma and succinate dehydrogenase‐deficient renal cell carcinoma.

**Abbreviations & Acronyms**
CT: computed tomography
FDG: fluorodeoxyglucose
HIF: hypoxia inducible factor
IVC: inferior vena cava
MIBG: metaiodobenzylguanidine
PET: positron emission tomography
RCC: renal cell carcinoma
SDH: succinate dehydrogenase

**Keynote message**

We reported a case of succinate dehydrogenase‐deficient malignant paraganglioma complicated by succinate dehydrogenase‐deficient renal cell carcinoma. Both tumors derived from the identical gene mutation and showed increased uptake of fluorodeoxyglucose due to the facilitated anaerobic metabolism. The former has a high risk for malignant behavior, while the latter is generally thought to have favorable prognosis.

## Introduction

Several genes are known to be responsible for the development of pheochromocytoma/paraganglioma.^1^ Of these, gene mutation of *SDH* is one of the most common, and this gene is also well known as a gene responsible for RCC.

SDH has four subunits: SDH‐A, ‐B, ‐C, and ‐D. Of these, mutation of the *SDHB* gene is the most frequently observed, and is thought to be associated with malignant potential of pheochromocytoma/paraganglioma.^2^ Here, we report a case of SDH‐deficient malignant paraganglioma complicated by SDH‐deficient RCC.

## Case report

**Fig. 1:**
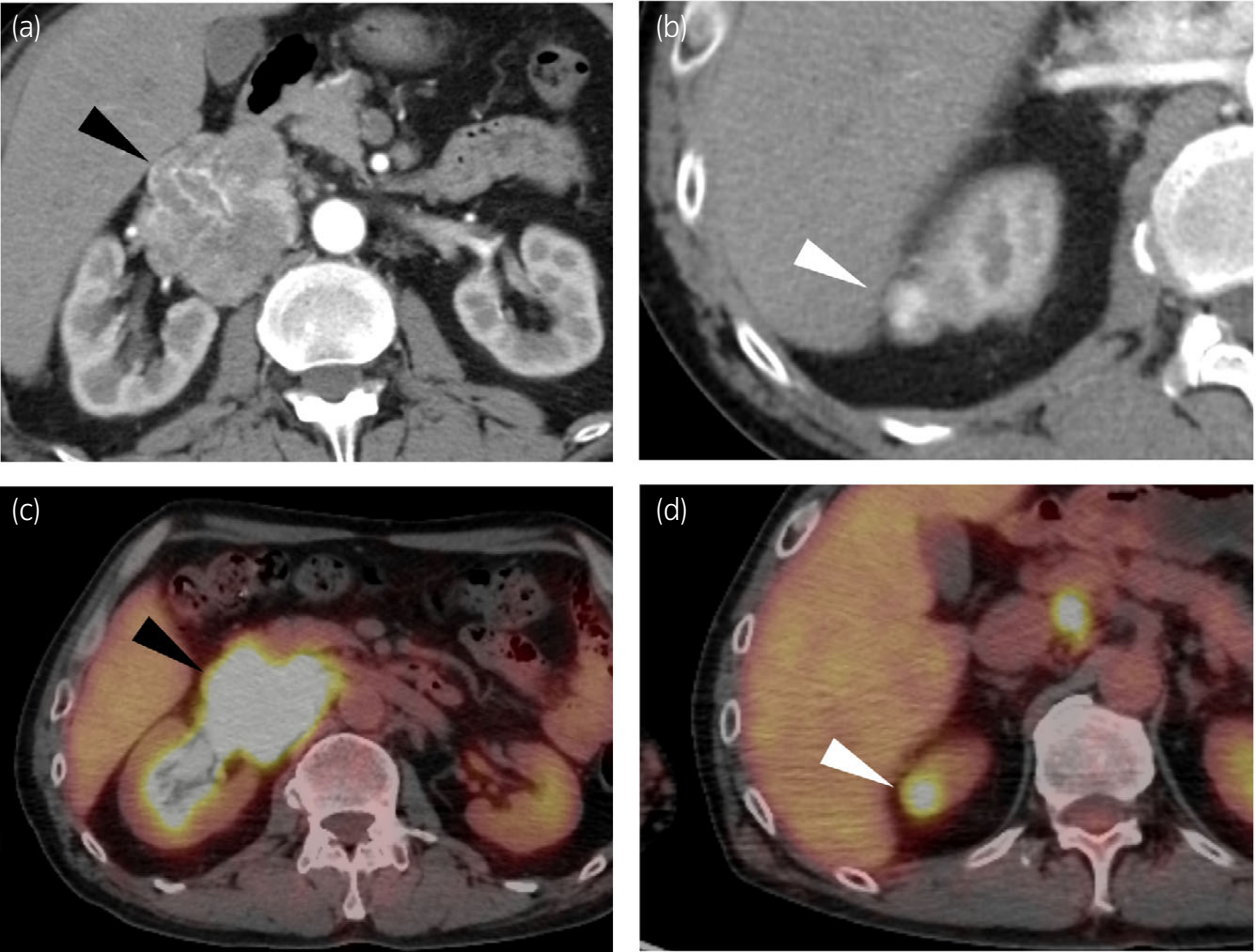
Radiological findings. Contrast‐enhanced CT images of (a) retroperitoneal paraganglioma (black arrowhead) and (b) right renal tumor (white arrowhead); ^18^F‐FDG‐PET images of (c) retroperitoneal paraganglioma (black arrowhead) and (d) right renal tumor (white arrowhead).

A retroperitoneal tumor was incidentally pointed out by ultrasonography at a medical checkup in a 50‐year‐old male. The patient was taking antihypertensive medication and had no familial history of malignant disease or endocrinological disease. Contrast‐enhanced CT showed a 6 cm retroperitoneal tumor located behind the IVC (Fig. 1a) and a 1 cm renal tumor at the upper pole of the right kidney (Fig. 1b). On ^18^F‐FDG‐PET, both tumors exhibited an increased uptake of FDG (Fig. 1c,d).

Endocrinological tests showed high plasma and urine levels of catecholamines. On ^123^I‐ MIBG scintigraphy, abnormal uptake was only observed in the right retroperitoneal tumor (Fig. S1).

Following the diagnosis of retroperitoneal paraganglioma with renal metastasis or primary RCC, en bloc resection of the retroperitoneal tumor, IVC, and right kidney, and IVC replacement with a prosthetic graft were carried out.

**Fig. 2:**
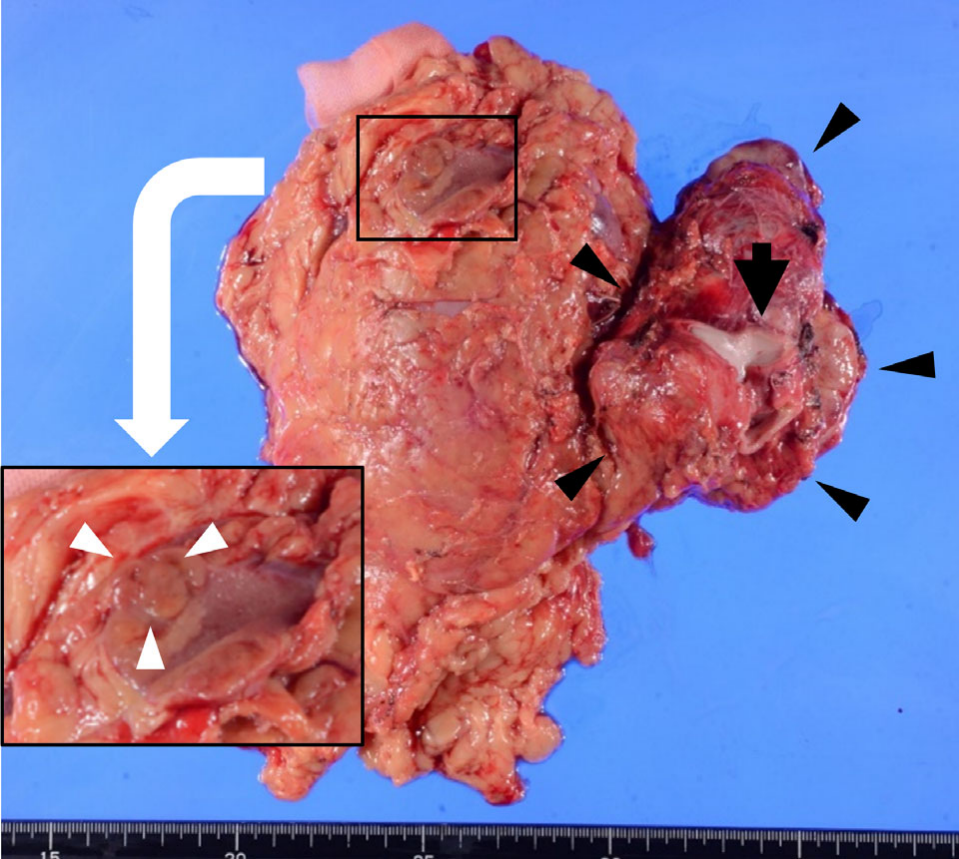
Macroscopic findings of the resected specimen; the retroperitoneal paraganglioma (black arrowhead), right renal tumor (white arrowhead), and inferior vena cava (black arrow).

**Fig. 3:**
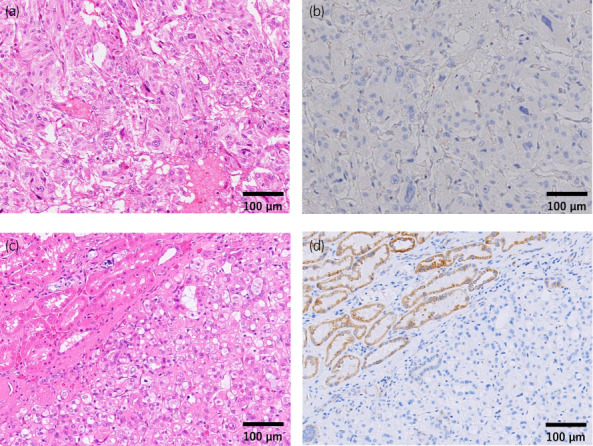
(a) Microscopic findings of the retroperitoneal paraganglioma, hematoxylin–eosin staining. (b) Paraganglioma was negative for immunostaining of SDH‐B. (c) Microscopic findings of the right renal tumor, hematoxylin–eosin staining. (d) The right renal tumor was negative for immunostaining of SDH‐B (lower part) although the normal renal parenchyma was positive (upper part).

Macroscopically, an 8 cm brown retroperitoneal tumor was contacting the right kidney, and a 1.5 cm yellow‐brown renal tumor was located on the upper pole of the right kidney (Fig. 2). Histological examinations revealed eosinophilic tumor cells with nuclear atypia and an alveolar proliferation pattern in the retroperitoneal paraganglioma (Fig. 3a), and the tumor cells invaded surrounding fat tissue, and IVC wall. The grading system for adrenal pheochromocytoma and paraganglioma score^3^ was 9, with a Ki‐67 labeling index of 22.5%, which indicated that the tumor was poorly differentiated and had a high risk for malignant behavior. The retroperitoneal paraganglioma was positive for chromogranin A and synaptophysin and negative for SDH‐B immunostaining (Fig. 3b). The right renal tumor had different histological features from the paraganglioma, and exhibited a solid proliferation pattern of eosinophilic cells with oval‐shaped nuclei (Fig. 3c). In immunostaining, the right renal tumor showed negative expression of SDH‐B (Fig. 3d), and chromogranin A and synaptophysin were negative. The right renal tumor was diagnosed as SDH‐deficient RCC, and its low proliferative potential was indicated by the Ki‐67 labeling index of <1%.

**Fig. 4:**
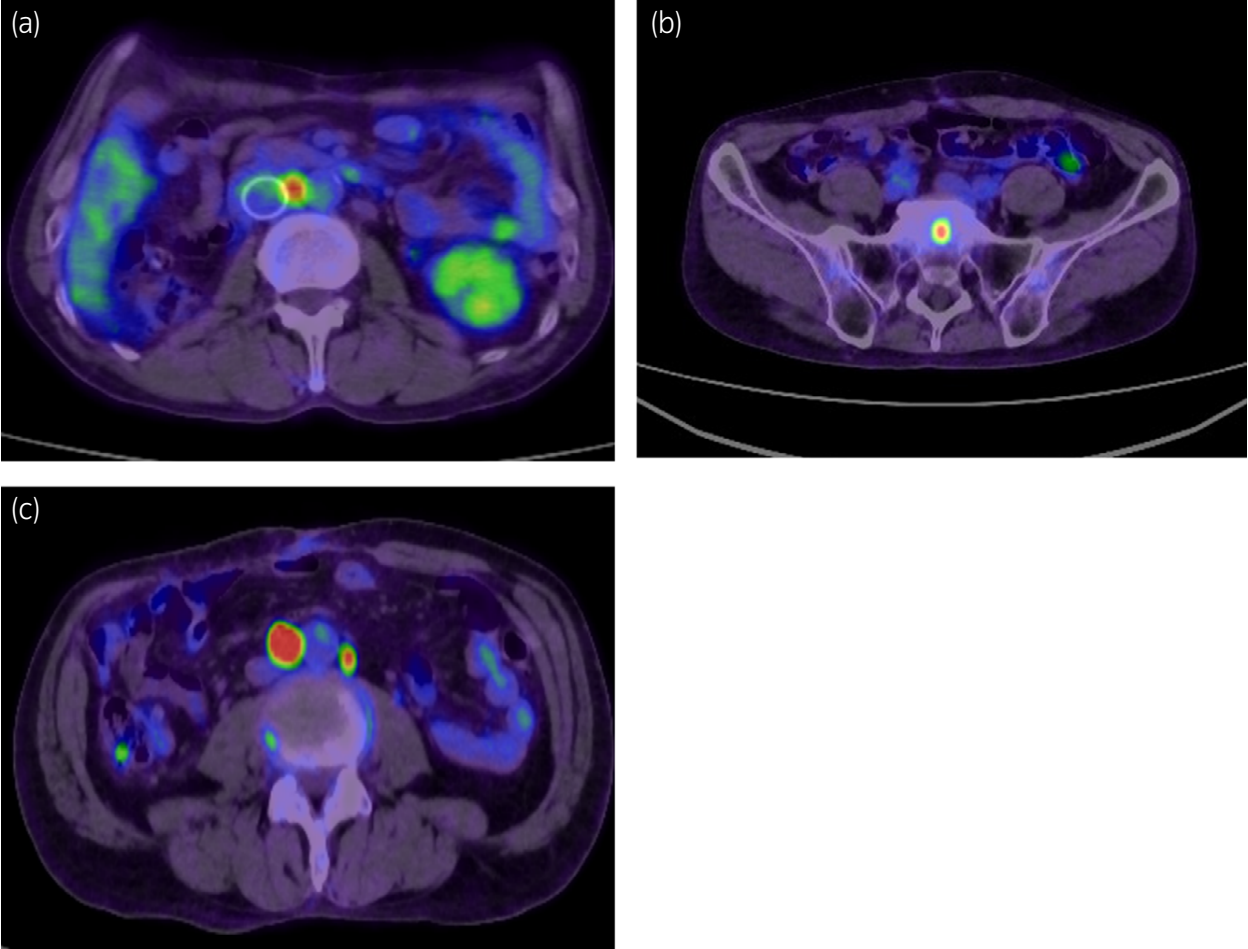
^18^F‐FDG‐PET showed (a) retroperitoneal lymph node metastasis 1 year after the first surgery, and (b) sacrum metastasis and (c) multiple retroperitoneal lymph node metastases 4 months after the second surgery.

Twelve months after surgery, a growing retroperitoneal lymph node was detected on CT. ^18^F‐FDG‐PET demonstrated an increased uptake of the node (Fig. 4a), although ^123^I‐ MIBG scintigraphy did not show any abnormal accumulation. Five months later, the patient underwent lymphadenectomy, which revealed lymph node metastasis of the paraganglioma. On initial follow‐up CT after the second surgery, multiple retroperitoneal lymph node swelling was observed. ^18^F‐FDG‐PET indicated sacral bone metastasis in addition to multiple lymph node metastases (Fig. 4b, c). Genome sequencing of the resected lymph node was carried out to determine the optimal treatment, and revealed the deletion of the *SDHB* gene. The mutation of *SDHB* gene in blood was confirmed by subsequent genetic blood testing, which indicated the germline mutation of *SDHB* gene. Thereafter, antiangiogenic therapy with sunitinib was initiated, and the patient maintained stable disease for 5 months.

## Discussion

We reported a case of malignant paraganglioma complicated by RCC, both of which were germinated by the identical gene mutation of *SDHB* deletion. To our knowledge, this is the first report of a case of synchronously diagnosed SDH‐deficient paraganglioma and SDH‐deficient RCC.

Deficiency of SDH, which converts succinate to fumarate, inhibits the citric acid cycle, and results in induction of HIF by facilitating anaerobic metabolism.^4^, ^5^ Through the excessive induction of HIF, *SDH* gene mutation can be responsible for the tumorigenesis of both pheochromocytoma/paraganglioma and RCC.^6^ In the present case, the increasing uptakes of FDG in both tumors reflected the increasing demand for sugar due to the facilitated anaerobic metabolism.^7^

It is reported that deletion of the *SDH* gene is involved in 10 to 30% of cases of pheochromocytoma/paraganglioma, and its penetrance at age 50 is estimated to be approximately 50%.^8^ Specifically, mutation of *SDHB* is considered to be highly associated with malignant behavior of pheochromocytoma/paraganglioma.^2^ When SDH‐B deficiency is observed in patients with hereditary pheochromocytoma/paraganglioma, the risks of metastasis and recurrence are reported to be 30 to 70%.^9^ In the present case, consistent with the genetic background, the grading system for adrenal pheochromocytoma and paraganglioma score was 9 out of a maximum score of 10, and metastasis developed postoperatively.

SDH‐deficient RCC, the entity of which was established in the 2016 World Health Organization Classification,^10^ is very rare and accounts for around 0.1% of all RCCs. In patients with SDH‐deficient RCC, deletion of *SDHB* is the most commonly observed mutation. SDH‐deficient RCC is generally thought to have favorable prognosis,^11^ while the presence of necrosis, high grade, and sarcomatoid features predicts worse prognosis.^12^ In the present case, the renal cancer would have less aggressiveness than the paraganglioma because of the low proliferative potential and the absence of the adverse features.

Due to the rarity of the disease, the treatment strategy for malignant pheochromocytoma/paraganglioma has not yet been established. The efficacy of conventional systemic chemotherapy is limited.^13^ Recently, several researchers have reported on the efficacy of antiangiogenic agents such as sunitinib.^14^ In SDH‐deficient pheochromocytoma/paraganglioma, deficiency of SDH results in stabilization and activation of the HIF‐1α, which promotes cancer growth due to the upregulation of angiogenic factors. Therefore, antiangiogenic therapy can be a treatment option for malignant pheochromocytoma/paraganglioma.

## Conclusion

We reported a rare case of SDH‐deficient malignant paraganglioma complicated by SDH‐deficient RCC. Although both tumors derived from the identical gene mutation and showed increased uptake of FDG due to the facilitated anaerobic metabolism, their biological behaviors were different.

## Author contributions

Yoshitomo Yamaguchi: Writing – original draft. Minato Yokoyama: Supervision; writing – review and editing. Akira Takemoto: Writing – review and editing. Yuki Nakamura: Writing – review and editing. Shohei Fukuda: Writing – review and editing. Sho Uehara: Writing – review and editing. Hajime Tanaka: Writing – review and editing. soichiro yoshida: Writing – review and editing. Yoh Matsuoka: Writing – review and editing. Yasuhisa Fujii: Writing – review and editing.

## Conflict of interest

The authors declare no conflict of interest.

## Approval of the research protocol by an Institutional Reviewer Board

Not applicable.

## Informed consent

Informed consent was obtained from the participant included in the study.

## Registry and the Registration No. of the study/trial

Not applicable.

## Supporting information


**Fig. S1** ^123^I‐MIBG scintigraphy only demonstrated abnormal uptake in the right retroperitoneal tumor (black arrowhead).
