# Supplementary figures and images for: Succinate dehydrogenase‐deficient malignant paraganglioma complicated by succinate dehydrogenase‐deficient renal cell carcinoma
Source: IJU Case Rep. 2022 Aug 3;5(6):480–3. doi: 10.1002/iju5.12520 (PMC9626355; doi:10.1002/iju5.12520)

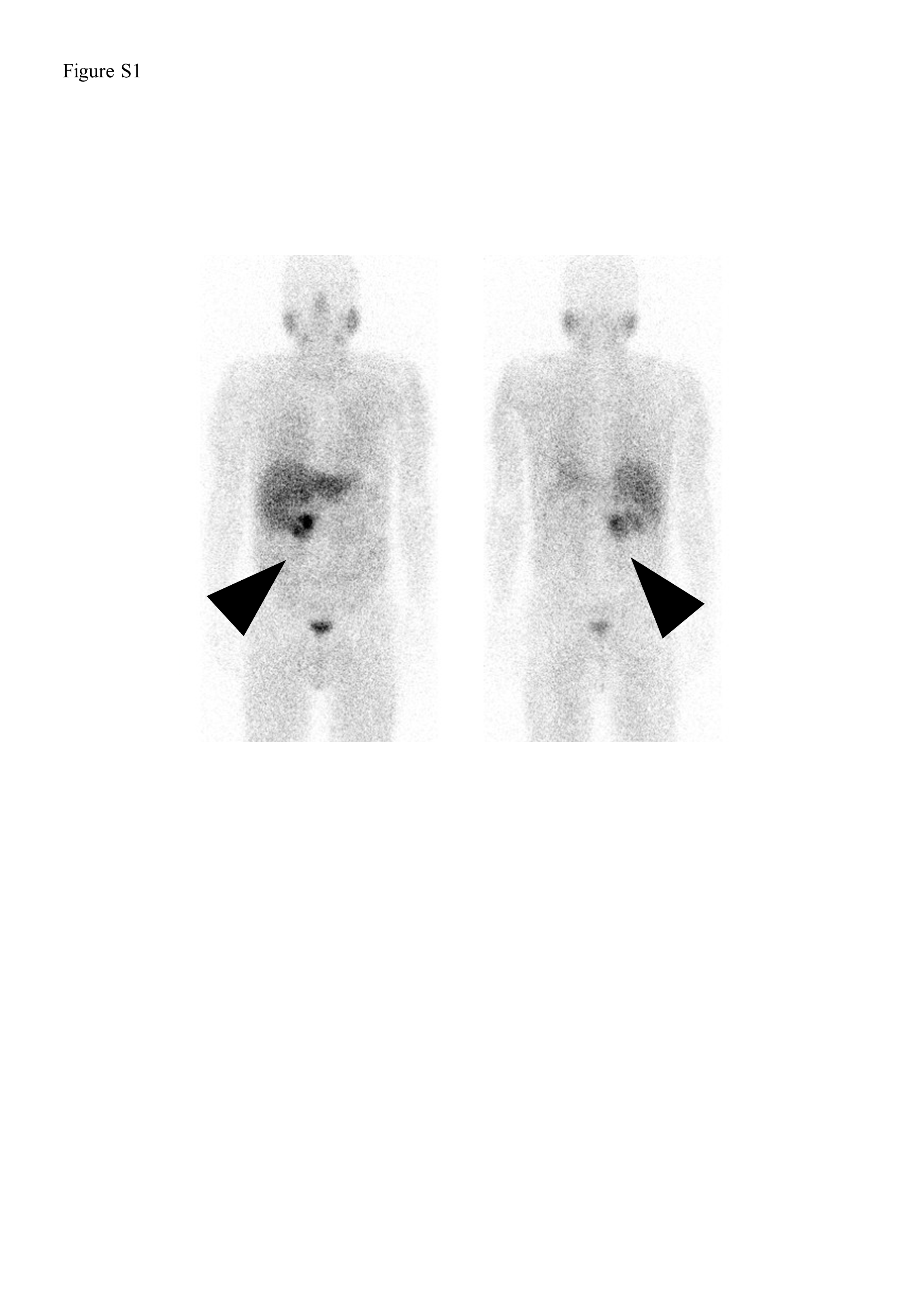

Supplement: Supplementary file 1 — Fig. S1 123I‐MIBG scintigraphy only demonstrated abnormal uptake in the right retroperitoneal tumor (black arrowhead). [file IJU5-5-480-s001.TIF]
